# Prefrontal tDCS Decreases Pain in Patients with Multiple Sclerosis

**DOI:** 10.3389/fnins.2016.00147

**Published:** 2016-04-08

**Authors:** Samar S. Ayache, Ulrich Palm, Moussa A. Chalah, Tarik Al-Ani, Arnaud Brignol, Mohamed Abdellaoui, Dalia Dimitri, Marc Sorel, Alain Créange, Jean-Pascal Lefaucheur

**Affiliations:** ^1^EA 4391, Excitabilité Nerveuse et Thérapeutique, Université Paris-Est-CréteilCréteil, France; ^2^Service de Physiologie – Explorations Fonctionnelles, Hôpital Henri Mondor, Assistance Publique – Hôpitaux de ParisCréteil, France; ^3^Neurology Division, Lebanese American University Medical Center - Rizk HospitalBeirut, Lebanon; ^4^Department of Psychiatry and Psychotherapy, Ludwig-Maximilian-UniversityMunich, Germany; ^5^Département d'Informatique et de Recherche Opérationnelle, Université de MontréalMontréal, QC, Canada; ^6^Service de Neurologie, Hôpital Henri Mondor, Assistance Publique – Hôpitaux de ParisCréteil, France

**Keywords:** pain, attention, multiple sclerosis, transcranial direct current stimulation, dorsolateral prefrontal cortex

## Abstract

**Background:** In the last few years, transcranial direct current stimulation (tDCS) has emerged as an appealing therapeutic option to improve brain functions. Promising data support the role of prefrontal tDCS in augmenting cognitive performance and ameliorating several neuropsychiatric symptoms, namely pain, fatigue, mood disturbances, and attentional impairment. Such symptoms are commonly encountered in patients with multiple sclerosis (MS).

**Objective:** The main objective of the current work was to evaluate the tDCS effects over the left dorsolateral prefrontal cortex (DLPFC) on pain in MS patients.Our secondary outcomes were to study its influence on attention, fatigue, and mood.

**Materials and Methods:** Sixteen MS patients with chronic neuropathic pain were enrolled in a randomized, sham-controlled, and cross-over study.Patients randomly received two anodal tDCS blocks (active or sham), each consisting of three consecutive daily tDCS sessions, and held apart by 3 weeks. Evaluations took place before and after each block. To evaluate pain, we used the Brief Pain Inventory (BPI) and the Visual Analog Scale (VAS). Attention was assessed using neurophysiological parameters and the Attention Network Test (ANT). Changes in mood and fatigue were measured using various scales.

**Results:** Compared to sham, active tDCS yielded significant analgesic effects according to VAS and BPI global scales.There were no effects of any block on mood, fatigue, or attention.

**Conclusion:** Based on our results, anodal tDCS over the left DLPFC appears to act in a selective manner and would ameliorate specific symptoms, particularly neuropathic pain. Analgesia might have occurred through the modulation of the emotional pain network. Attention, mood, and fatigue were not improved in this work. This could be partly attributed to the short protocol duration, the small sample size, and the heterogeneity of our MS cohort. Future large-scale studies can benefit from comparing the tDCS effects over different cortical sites, changing the stimulation montage, prolonging the duration of protocol, and coupling tDCS with neuroimaging techniques for a better understanding of its possible mechanism of action.

## Introduction

Multiple sclerosis (MS) is a chronic progressive inflammatory disease of the central nervous system, and represents the major cause of non-traumatic disability in young adults (Noseworthy et al., 2000; Compston and Coles, 2008). During the course of the disease, the patients may develop sensorimotor, cognitive, emotional, and behavioral symptoms. For instance, although MS was previously considered a painless disease, chronic pain has been recently reported as a debilitating symptom (Ehde et al., 2005), with a prevalence varying between 29 and 86% (O'Connor et al., 2008). The lower limbs dysesthesias remain the most common and difficult to treat painful symptoms, attributed to demyelination and axonal degeneration processes involving the central sensory pathways (O'Connor et al., 2008). Psychiatric comorbidities are also common (Marrie et al., 2015). In particular, anxiety and depression were found to occur in about 21.9 and 23.7% of MS patients, respectively (Marrie et al., 2015). Moreover, up to 75% of MS patients can face fatigue at some point during the disease course; such a symptom was found to have a drastic impact on their quality of life (Chalah et al., 2015). Furthermore, abnormalities in the attentional capacities have been described in MS patients (Urbanek et al., 2010; Crivelli et al., 2012; Omisade et al., 2012; Vázquez-Marrufo et al., 2014; Ayache et al., 2015).

The interaction between pain and attention has gained an increasing interest in the last decade. Although pain serves as a warning signal for responding to potential hazards, its relationship with the intensity of a stimulus is not linear. It is rather modulated by several factors, among which attention appears to play a major role (Van Damme et al., 2010). On the one hand, distraction was documented to decrease the perceived intensity of provoked pain (Legrain et al., 2002). On the other hand, pain can capture the attentional processes, in a way that prohibits good performance on cognitive tasks, even with a voluntary neglect of the nociceptive stimuli (Eccleston and Crombez, 1999; Legrain et al., 2009a,b; Van Damme et al., 2010). In this perspective, the neurocognitive model of attention to pain explains the aforementioned interaction by two modes of selection: the top-down attention, which prioritizes relevant information and prevents irrelevant stimuli such as pain from capturing attention, and the bottom-up attention by which unintentional stimulus captures the attention (Legrain et al., 2009a).

The current advances in neuroimaging and neurophysiological modalities have unveiled the key role of the dorsolateral prefrontal cortex (DLPFC) in the circuits of pain (Lorenz et al., 2003), fatigue (Chalah et al., 2015), depression (Gobbi et al., 2014), and attention (Petersen and Posner, 2012), including the attentional circuit dedicated to noxious stimuli (Legrain et al., 2009a). Among the available literature regarding experimental pain, one study has revealed negative correlations between the activation patterns within the DLPFC bilaterally and each of pain intensity and unpleasantness (Lorenz et al., 2003). Here, the DLPFC was thought to modulate pain perception through corticosubcortical and corticocortical pathways. In addition, DLPFC seems to be a major component of the so-called cortico-striato-thalamo-cortical fatigue loop in MS (Chalah et al., 2015). Moreover, the middle frontal gyrus, which embeds the DLPFC, was found to be selectively related to depression in MS (Gobbi et al., 2014). Furthermore, DLPFC constitutes a main component of the attentional circuits, particularly the fronto-parietal executive control network (Petersen and Posner, 2012). This was based on lesions studies in human where the DLPFC appears to be involved in switching from one set of tasks to the other (Petersen and Posner, 2012). Nevertheless, experimental research investigating the interaction between bottom-up and top-down attention has highlighted the role of DLPFC in processing the painful stimuli (Legrain et al., 2009a). By maintaining the attentional load, this cortical area prioritizes goal-relevant information in order to prevent the attentional capture by pain (Legrain et al., 2009a). Therefore, one can assume that acting on DLPFC might have an impact on pain perception, attentional resources, mood, and fatigue.

Noninvasive brain stimulation (NIBS) is currently being investigated for the management of neuropsychiatric symptoms when pharmacological interventions fail. Among those techniques, transcranial direct current stimulation (tDCS) has gained a particular interest in recent years, and appears to be a promising tool for the treatment of several neurological disorders. It acts by changing the cortical excitability (Nitsche and Paulus, 2000, 2001; Nitsche and Fregni, 2007; Nitsche et al., 2008), notably by depolarizing (activating) and hyperpolarizing (inhibiting) the cortical circuits, in the case of anodal and cathodal stimulation, respectively.

In the present work, we applied anodal tDCS over the left DLPFC. Our primary endpoint was to evaluate its effects on neuropathic pain in MS patients. Our secondary outcomes were to assess its impact on attention, mood, and fatigue.

## Materials and methods

### Ethics statement

This is a prospective, randomized, cross-over, sham-controlled study, conducted according to the declaration of Helsinki, and approved by the local ethical committee (CPP-Ile de France VI, registered as N° 2012-A00721-42). The trial is registered at the Deutsches Register Klinischer Studien (drks-neu.uniklinik-freiburg.de) and has the following registration number: DRKS00005296. All participants were well instructed about the protocol and voluntarily gave their written informed consent prior to inclusion.

### Study participants

Patients were enrolled by M.A., M.S., D.D., and A.C. from the Neurology department of Henri Mondor Hospital, Créteil, France, between November 2012 and November 2014 according to the following criteria: (i) a definite MS diagnosis according to the 2010 revised McDonald criteria (Polman et al., 2011); (ii) age between 18 and 70 years; (iii) right handedness based on the Edinburgh inventory (Oldfield, 1971); and (iv) a history of neuropathic pain since more than 3 months as per the Neuropathic Pain Symptom Inventory (NPSI; Bouhassira et al., 2004), with an intensity >40 on the visual analog scale from 0 to 100 (VAS_0−100_), obtained as the average of daily scores over a representative week.

Exclusion criteria consisted of the following: (i) MS relapses within the last 2 months; (ii) changes in pharmacological and physical therapies during the last month; (iii) the presence of comorbid neurodegenerative or psychiatric disorders; (iv) history of substance abuse; (v) absence of measurable pain related evoked potentials (PREPs) at the right hand; (vi) severe deficit in the visual acuity or fields as documented by an ophthalmic exam; and (vii) severe right upper limb impairment as per the Medical Research Council scale for muscle power (MRC) (Medical Research Council, 1981). For the latter, we applied the MRC score to the four muscle groups involved in pinching, wrist extension, forearm flexion, and arm abduction, so that the sum of their scores could vary between 0 (null strength) and 20 (full strength); an MRC score < 12 excluded the individual from participation.

A standard neurological examination was performed in all patients including a documentation of the disability level based on the expanded disability status scale (EDSS; Kurtzke, 1983), and a thorough pain evaluation.

### tDCS

**Figure 1:**
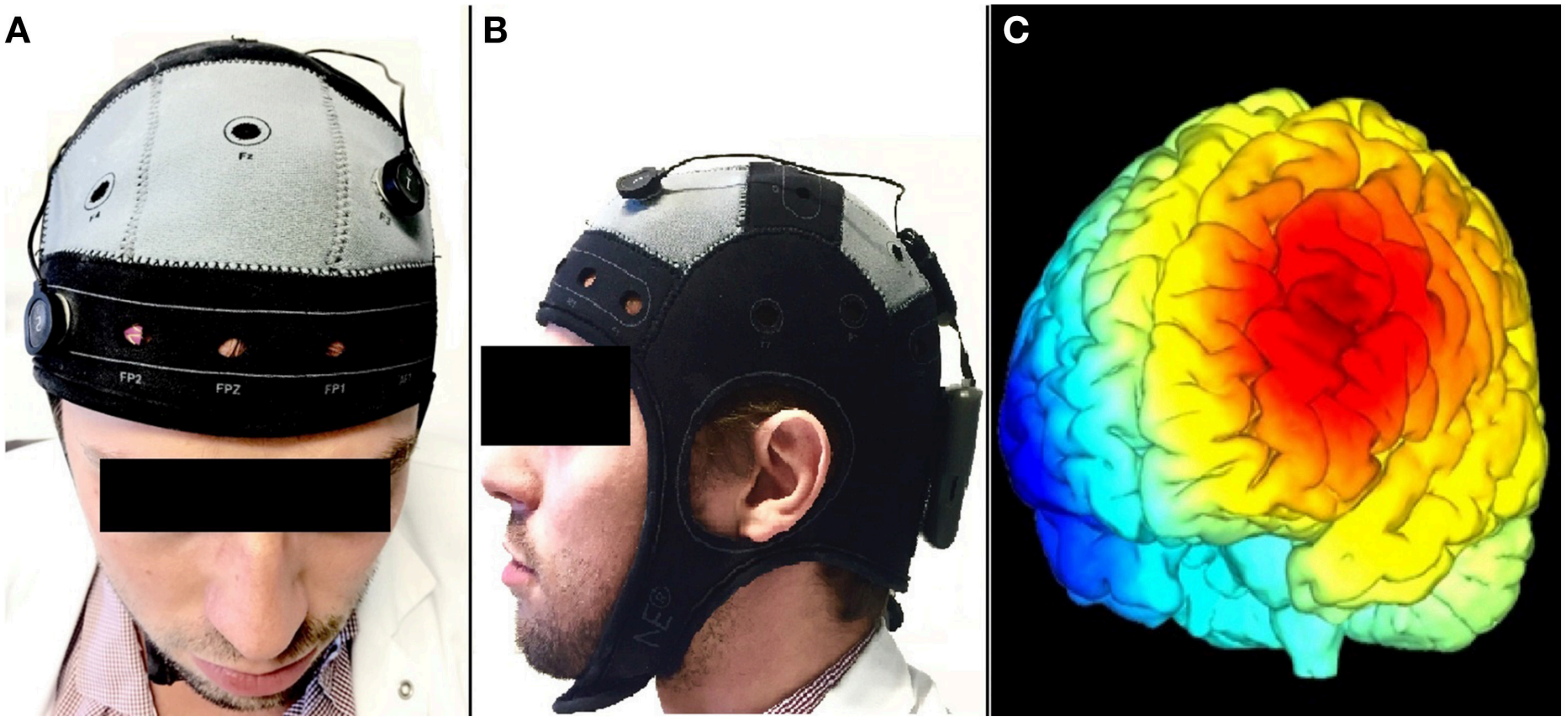
**An illustration of the tDCS montage in this study (Starstim, Neuroelectrics, Barcelona, Spain); with a cathode over AF8 (A), an anode over F3 (A,B) according to the international 10–20 EEG system**. A simulation of the electric field generated between F3 (in red) and AF8 (in blue) is shown in **(C)**.

A battery driven multi-channel direct current stimulator (Starstim, Neuroelectrics, Barcelona, Spain) delivered the direct current over the scalp through sponge electrodes (surface area = 25 cm^2^), soaked in a saline solution to minimize the risk of skin irritation (Palm et al., 2014). The stimulation electrodes were directly positioned on an adult sized cap worn by the patients, and labeled according to the 10–20 EEG system of electrode positioning (Starstim, Neuroelectrics, Barcelona, Spain). To stimulate the left DLPFC, the anode was placed over F3, and its corresponding cathode over the right supraorbital region (Figure 1). The used current intensity was 2 mA (total current density over the stimulated area: 0.06 mA/cm^2^) which is below the threshold for tissue damage (Poreisz et al., 2007; Nitsche et al., 2008). For the active stimulation, the current was ramped up during the first 15 s to a maximum of 2 mA that was maintained throughout the 20-min stimulation session. As for the sham stimulation, the current was ramped down immediately after ramping up in order to achieve an effective blinding (Gandiga et al., 2006; Ambrus et al., 2012).

Sham or active anodal tDCS blocks were tested in a random order and were held apart by at least 3 weeks. Each block consisted of three consecutive daily tDCS sessions. The stimulations were performed by well-trained physicians. The sessions took place in a quiet and illuminated room. The patients were at rest in an armchair and were not performing any cognitive task. Only the performing physician (S.S.A) was aware of the stimulation mode (real or sham tDCS). The evaluators (U.P and M.A.C) and the patients were blind to it.

### Primary outcomes: Pain scales

For pain evaluation, we relied on the self-reported VAS_0−100_ and the short version of the Brief Pain Inventory (BPI). The latter is a validated and reliable tool to measure pain intensity and its interference with patient's life (Cleeland and Ryan, 1994).

### Secondary outcomes

#### Cognitive task

Attention was evaluated using a computerized test: The Attention Network Test (ANT; Fan et al., 2002). Briefly, this test evaluates the three main attentional networks: the alerting network that consists of controlling vigilance and task performance, the orienting network responsible for the orientation to external stimuli, and the executive function network in charge of conflict resolution (Fan et al., 2002; Petersen and Posner, 2012). The test was performed in a quiet room, with the participants sitting in front of a monitor aligned to their midsagittal plane. The stimulus consists of a row of five horizontal black arrows, against a white background: a “central” arrow surrounded on each side by two others called “flankers” which can be pointing left- or right-ward. Several possible conditions arise: a “congruent” condition takes place when the flankers and the central arrow are in same direction; an “incongruent” condition occurs when the flankers and the central arrow are in opposite direction; and a “neutral” condition occurs when the flankers are replaced instead by four strokes. The participants indicated the direction of the central arrow by clicking on the left or right mouse buttons, with either the right index or the right middle finger, when the central arrowhead pointed to the left or right, respectively. During the total duration of the test, the subjects are asked to fix a central cross, and are informed about a warning “cue,” which consists of a star-shaped signal that may precede the stimulus in question, and thus might either help or not in localizing the latter. Here, four possibilities exist: “no cue” condition when there is no preceding signal, “center cue” condition when it appears at the center of the screen, “double cue” condition when signals are simultaneously provided above and below the central cross, and a “spatial” cue condition when the signal is presented either up or down the cross. We used the 1.3.0 version of ANT, which includes one practice sequence (24 trials), and three identical sequences of 96 trials each, separated by an optional resting period. The participants should answer correctly as fast as possible, and the test provides the mean accuracy (MA) and the mean reaction time (MRT), which reflect the errors rate and the time required by the participant to answer, respectively. Moreover, all conditions are analyzed to assess the integrity of the attentional networks. For instance, the alerting network is evaluated when the participant fixes the central cross; the orienting network is put into play when the warning signal is presented prior to the stimulus; and the executive function network is recruited when the subject is trying to solve the conflict and takes a decision in order to answer.

#### Neuropsychological assessment

Mood and fatigue were evaluated using the 14-item Hospital Anxiety and Depression Scale (HADS; Snaith and Zigmond, 1986) and the 21-item Modified Fatigue Impact Scale (MFIS), respectively (Fatigue guidelines development panel of the multiple sclerosis council for clinical practice guidelines, 1998; Téllez et al., 2005). MFIS accounts for the physical (9 items), cognitive (10 items), and psychosocial (2 items) components of fatigue.

#### Neurophysiological testing

##### Pain related evoked potentials (PREPs)

PREPs can be generated at the cortical level by a variety of noxious stimuli (Kakigi et al., 2005; Arendt-Nielsen and Andersen, 2006), namely the lasers (Arendt-Nielsen and Chen, 2003). Their amplitudes serve as physiological correlates for the amount of attention paid toward noxious stimuli, and reflect the functionality of the involved neural networks (Garcia-Larrea et al., 2002; Lorenz and Garcia-Larrea, 2003). A concentric planar electrode—which was previously found to be an alternative to laser in inducing PREPs—has served the purpose in this work (Kaube et al., 2000; Lefaucheur et al., 2012).

The first step consisted of defining the pain perception threshold (PPT). The PPT was determined by the method of limits, which consists of raising the stimulus intensity from zero to the point where the stimulus is perceived as painful sensation at intensity of 60–70 on VAS_0−100_. The threshold was determined three times. The average of the three trials was defined as the PPT.

Stimulation was carried out using a concentric electrode designed to excite the superficial skin layers, hence the nociceptive axons (Lefaucheur et al., 2012). Stimuli were applied at the first dorsal interosseous space of the right hand, the cutaneous area located on the dorsal and lateral aspect of the hand between the extensor pollicis longus tendon and the extensor indicis tendon. A single stimulus was a train of three pulses that lasts 0.5 ms, with an inter-pulse interval (IS) of 5 ms.

After determining the PPT, two sets of twenty stimuli were performed at irregular intervals, at VAS_60−70._The PREPs were recorded via 10 mm Genuine Grass Gold Cup Electrodes (Grass Products, Astro-Med, Inc., Natus Neurology, Warwick, RI 02886 U.S.A.), fixed to the scalp surface using a special paste (Grass Products, Astro-Med, Inc., Natus Neurology, Warwick, RI 02886 U.S.A.). The electrodes were positioned as the following: the recording one at Cz (international 10–20 EEG system of electrode positioning), the reference one at the left earlobe, and the ground one with a Velcro strap around the right forearm (Ref NT-S07ALPINE ground electrode with Velcro Strap, Alpine Biomed, Skovlunde, Denmark). In addition, electro-oculogram was obtained via pre-gelled adhesive surface electrodes (Ref 9013S0242, Alpine Biomed, Skovlunde, Denmark) placed at the right infraorbital lateral margin.

The recorded signal was bandpass filtered at 0.5–30 Hz and stored for analysis using a Keypoint machine (Dantec Dynamics, Bristol, United Kingdom). The final step consisted of averaging the signals offline, and excluding the contaminated recordings (saccadic eye movements or blinks, and raw signals above 70 V). We then analyzed the collected N2-P2 responses and measured their baseline-to-peak amplitudes, peak-to-peak amplitudes, and latencies.

##### Frontal midline theta activity

The frontal midline theta (FmΘ) is an oscillatory activity in the theta band (4–7 Hz) that appears in the medial frontal region at any time during the performance of a cognitive task, reaching 6–7 Hz in frequency and 30–60 mV in amplitude (Ishihara and Yoshi, 1972; Mizuki et al., 1980). We looked for this activity by recording EEG during the second ANT sequence. The recording electrodes were located on the patients' cap at Fpz and Fz (according to the international 10–20 EEG system of electrode positioning), and connected to the recording device (Starstim, Neuroelectrics, Barcelona, Spain). The raw EEG signals were stored for offline processing and analysis. These signals were then pass-band-filtered in the frequency range [0.5–30 Hz] to keep only the rhythms of interest delta, theta, alpha and beta.

**Figure 2:**
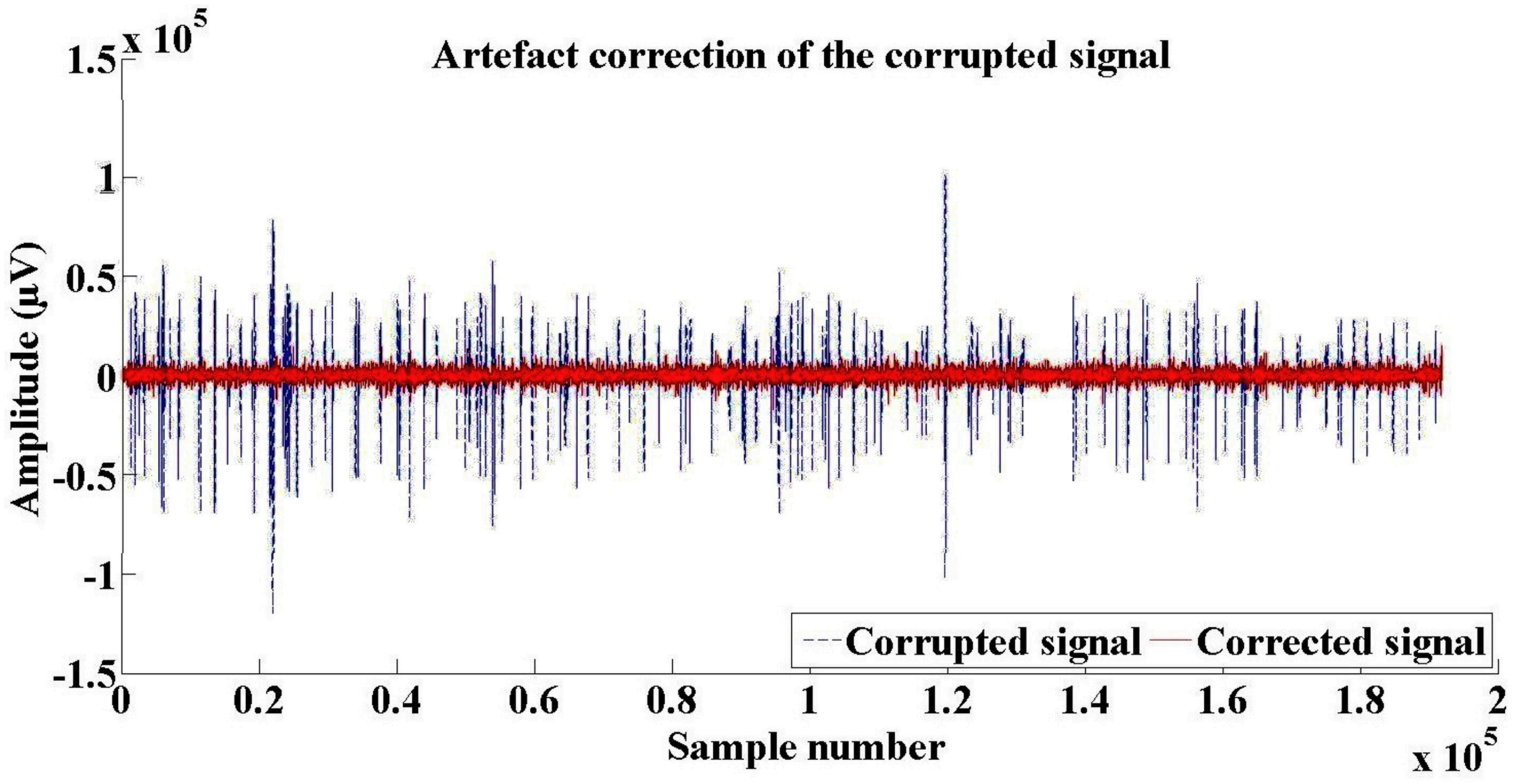
**An example of artifact rejection in a single EEG record**.

An important step before doing any analysis would be to reject ocular and blink artifacts that are highly abundant in raw data. For this, prior to any analysis, we applied on all data an algorithm that detects, localizes, and rejects artifacts in a single-channel biosignal. An example of the artifact rejection is shown in Figure 2.

##### EEG analysis

EEG analysis was performed by T.A. and A.B. To obtain a good estimate of the percentage held by the dominant rhythms (delta, theta, alpha, and beta) in a given raw EEG signals we determined the frequencies that dominate each signal.

First, the signals were band-pass filtered in the frequency bands [0.5–3.5 Hz], [4–7 Hz], [8–12 Hz], and [13–30 Hz] corresponding to the rhythms delta, theta, alpha, and beta respectively. To take into account the difference in the amplitudes of these rhythms, we determined the gradient of their amplitudes, looked for their envelopes, and calculated the modulus of the Hilbert transform of the gradients in question (Marple, 1999). At each sample of the given EEG signal, we selected the maximum modulus. Finally, the percentage of each frequency band in the given EEG signal of each patient was determined.

### Experimental protocol

**Figure 3:**
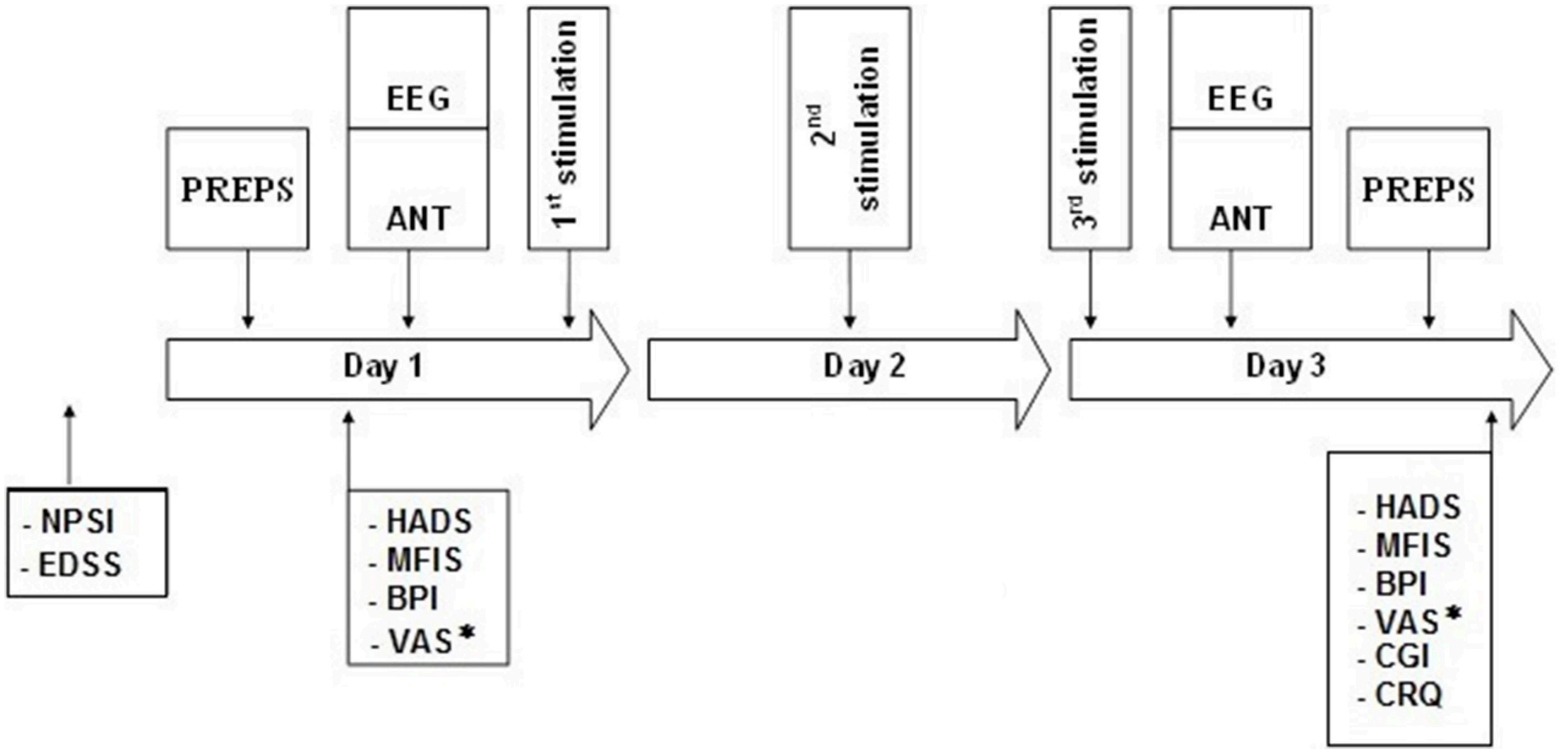
**Schematic diagram of the experimental protocol**. Two tDCS blocks, each consisting of 3 consecutive daily stimulations of either sham tDCS or active tDCS (randomized order); held apart by a 3-week interval.ANT, Attention Network Test; BPI, Brief Pain Inventory; CGI, Clinical Global Impression; CRQ, Comfort Rating Questionnaire; EEG, recording at Fpz and Pz during ANT; EDSS, Expanded Disability Status Scale; HADS, Hospital Anxiety and Depression Scale; MFIS, Modified Fatigue Impact Scale; NPSI, Neuropathic Pain Symptoms Inventory; PREPs, Pain Related Evoked Potentials; VAS^*^, Visual Analog Scale for pain assessed 7 days prior to the first stimulation session (D1) and 7 days after the last stimulation session (D3) of each block.

After the inclusion session, patients were randomized to receive either sham or active tDCS block (Figure 3). The randomization schedule was generated by U.P. prior to the beginning of the study using a dedicated software (“true” random number generation without any restriction, stored in a computer until the patient was assigned to the intervention).

Keeping in mind the fluctuating nature of neuropathic pain, and the effects of circadian rhythm on attentional capacities, the patients underwent the experimental protocol at the same time of the day (Knight and Mather, 2013). They were also asked to restrict stimulants consumption, like caffeine or nicotine, at least 3 h prior to their appointment. No blocks were performed during summer time to avoid any influence of temperature on fatigue (Chalah et al., 2015).

The patients daily recorded the average of their pain intensity 1 week before and 1 week after each block using VAS_0−100_. At the first day of each block (D1), they were asked to fill questionnaires for pain (BPI), fatigue (MFIS), anxiety and depression (HADS). This was followed by PREPs recording, the performance of ANT test, and finally the tDCS session. The latter was repeated at the second (D2) and third (D3) day of each block. At D3, the above-mentioned evaluations were repeated following the stimulation session, and the patients' degree of satisfaction regarding the protocol was assessed using the Comfort Rating Questionnaire (CRQ) and the Global Clinical Impression (GCI). At the end of each block, the patients were asked to guess the mode of stimulation (active/sham). A schematic diagram is shown in Figure 3.

### Data analysis

For the ANT parameters, we excluded from the analysis the reaction times of trials with either error or with a reaction time value above or below the mean ± 2 standard deviations (SD). Statistical analyses were performed using the StatView and InStat software. Our Data followed normal distributions according to the method of Kolmogorov and Smirnov. Data collected before and after each block were analyzed with paired *t*-tests, and those with a *p* < 0.05 were considered significant. This included PREPs (amplitudes and latencies), ANT parameters (MA and RT), frontal theta activity percentages, and self-rating questionnaires scores for pain (VAS_0−100_, BPI), fatigue (MFIS), anxiety and depression (HADS).

## Results

### Demographic and clinical data

**Figure 4:**
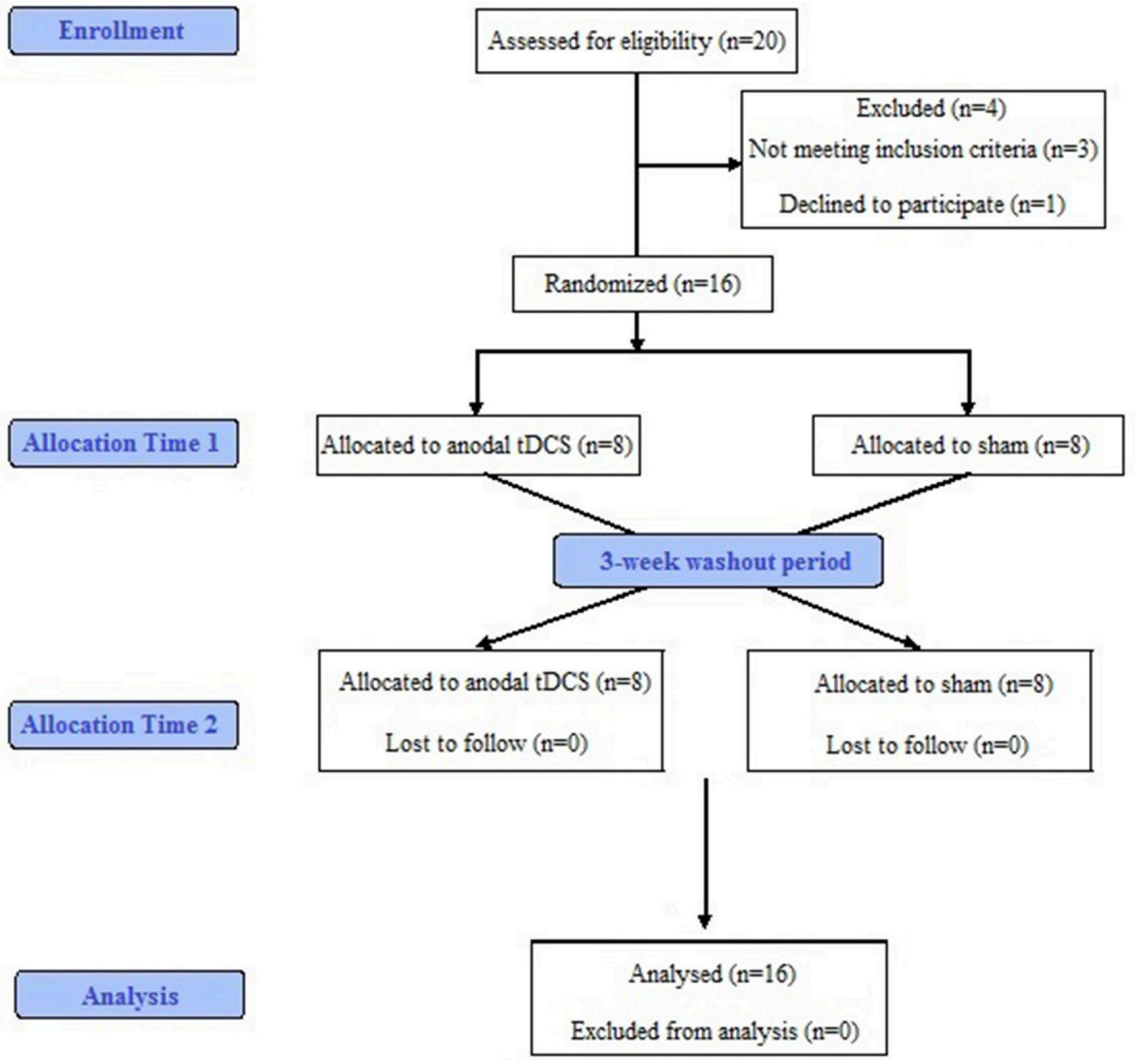
**Schematic flow chart of patients through the study**.

Twenty MS patients were enrolled, and four were excluded (one declined to participate, and three were not eligible). Sixteen patients (13 women, 3 men, mean age 48.9 ± 10.0 years, age range 38–67 years) completed the study (Figure 4 illustrates the flowchart of all participants). All had neuropathic pain; their mean NPSI was 5.12 ± 2.4 at enrollment. Their mean EDSS was 4.25 ± 1.4. Eleven patients had relapsing remitting MS (RRMS), four were in the secondary progressive phase (SPMS) and one had a primary progressive type (PPMS). The mean disease duration was 11.8 ± 9.4 years and the progressive phase duration was 6 ± 3.2 years.

All patients had concomitant medication intake, consisting of antiepileptic drugs (nine patients; one of them with two drugs), antidepressants (nine patients, one of them with two drugs), opioids (five patients, one of them with two drugs) and immunomodulating agents (13 patients). Pharmacological treatment was continued and kept stable throughout the experimental protocol.

### Adverse effects, comfort rating, and blinding integrity

After both active and sham stimulation, six patients reported insomnia. Nausea was reported by five patients after active stimulation and by four patients after sham stimulation. Severe headache occurred in three patients after active stimulation and in one patient after sham stimulation. Phosphenes were reported by one patient after sham stimulation.

No significant difference was found between both conditions regarding the CRQ sum scores during (*p* = 0.96) and after stimulation (*p* = 0.43). No significant differences were observed between both stimulation conditions on the overall discomfort (*p* = 0.79) or the mean GCI (*p* = 0.82; Friedman test). Details are provided in Table 1.

**Table 1:** **Confort rating questionnaire (CRQ) and global clinical impression (GCI) for sham and active tDCS**.

	**Sham**	**tDCS**	**paired *t*-test**
CRQ sum scores during stimulation	18.3 ± 12.3	19.1 ± 11.5	*P = 0.96*
CRQ sum scores after stimulation	20.7 ± 13.8	17.5 ± 9.6	*P = 0.43*
Overall discomfort after stimulation	2.4 ± 2.0	2.6 ± 2.3	*P = 0.79*
Mean GCI after stimulation	4.6 ± 0.8	4.6 ± 0.9	*P = 0.82*

### Pain perception

The mean VAS_0−100_ pain ratings 7 days before and 7 days after stimulation showed significant decrease after active tDCS (*p* = 0.024), but no change after sham tDCS (*p* = 0.66). Analogously, the mean VAS_0−100_ pain ratings for days 1–3 before and after stimulation showed significant decrease after active tDCS (*p* = 0.021), and no improvement after sham tDCS (*p* = 0.56).

Active stimulation resulted in a significant improvement of BPI global score (*p* = 0.02), and its interference subscale (*p* = 0.01), but had no significant effects on the severity subscale. Sham did not have any significant effects on BPI scores or its subscales. Pain scores are found in Table 2.

**Table 2:** **VAS and BPI scores (Mean ± SD) before and after sham and active tDCS**.

	**Before sham**	**After sham**	**Paired *t*-test**	**Before tDCS**	**After tDCS**	**Paired *t*-test**
VAS mean/7days	52.1 ± 19.6	50.3 ± 19.7	*P = 0.66*	51.2 ± 19.2	43.1 ± 26.2	*P = 0.02*
VAS mean/Day 1–3	48.8 ± 22.0	51.3 ± 18.8	*P = 0.56*	53.1 ± 20.2	43.1 ± 26.2	*P = 0.02*
BPI global score	9.9 ± 3.5	9.2 ± 3.4	*P = 0.19*	9.2 ± 3.4	8.2 ± 3.5	*P = 0.02*
BPI severity subscale	4.8 ± 2.4	4.6 ± 2.1	*P = 0.40*	4.8 ± 2.4	4.3 ± 2.1	*P = 0.15*
BPI interference subscale	5.0 ± 1.5	4.6 ± 1.6	*P = 0.18*	4.5 ± 1.6	3.9 ± 1.6	*P = 0.01*

### Cognitive results

There were no significant changes in any of the ANT parameters after both sham and active stimulation (Table 3).

**Table 3:** **ANT results (Mean ± SD) before and after sham and active tDCS**.

	**Before sham**	**After sham**	**Paired *t*-test**	**Before tDCS**	**After tDCS**	**Paired *t*-test**
ANT alertness	61.7 ± 60.5	58.8 ± 66.0	*P = 0.78*	36.5 ± 42.0	52.1 ± 36.0	*P = 0.08*
ANT orientation	53.7 ± 33.2	53.4 ± 27.5	*P = 0.98*	41.0 ± 30.5	50.2 ± 27.4	*P = 0.33*
ANT conflict	143.0 ± 67.9	141.8 ± 53.2	*P = 0.95*	143.1 ± 75.8	153.9 ± 69.0	*P = 0.43*
ANT mean reaction time	768.3 ± 95.0	766.1 ± 130.3	*P = 0.94*	725.4 ± 93.8	742.3 ± 99.7	*P = 0.38*
ANT accuracy	86.1 ± 21.7	90.2 ± 14.6	*P = 0.43*	92.4 ± 9.2	88.4 ± 15.0	*P = 0.17*

### Neuropsychological scores

The baseline mean global MFIS score, HADS anxiety and depression subscales were 52.6 ± 12.2, 9.2 ± 4.0, and 6.6 ± 3.2, respectively.

No significant differences were observed following both stimulation conditions for the MFIS total scores, HADS total scores and subscales. The neuropsychological scores are detailed in Table 4.

**Table 4:** **Neuropsychological results (Mean ± SD) before and after sham and active tDCS**.

	**Before sham**	**After sham**	***t*-test**	**Before tDCS**	**After tDCS**	***t*-test**
Mean HADS total score	14.4 ± 5.9	14.5 ± 6.5	*P = 0.80*	14.1 ± 6.3	13.6 ± 5.8	*P = 0.52*
Mean HADS anxiety	8.1 ± 3.4	8.3 ± 3.9	*P = 0.70*	7.7 ± 3.0	7.6 ± 3.6	*P = 0.90*
Mean HADS depression	6.3 ± 3.0	6.2 ± 3.3	*P = 0.81*	6.4 ± 3.9	6.0 ± 3.3	*P = 0.35*
MFIS global score	48.2 ± 15.5	47.4 ± 17.7	*P = 0.76*	49.5 ± 14.4	49.0 ± 15.2	*P = 0.42*

### Neurophysiological testing

#### PREPs

PREPs results did not significantly differ between active and sham stimulation (Table 5).

**Table 5:** **Neurophysiological (EEG and PREPS) results (Mean ± SD) before and after sham and active tDCS**.

	**Before sham**	**After sham**	**Paired *t*-test**	**Before tDCS**	**After tDCS**	**Paired *t*-test**
Theta Fpz	51.1 ± 14.6	58.9 ± 10.2	*P = 0.09*	53.8 ± 13.9	62.1 ± 11.1	*P = 0.09*
Theta Fz	53.9 ± 9.8	59.3 ± 7.9	*P = 0.04*	54.9 ± 15.1	62.6 ± 10.5	*P = 0.09*
Mean N2 latency	118.9 ± 34.8	131.7 ± 25.6	*P = 0.16*	138.1 ± 44.6	141.8 ± 33.2	*P = 0.66*
Mean P2 latency	178.0 ± 48.8	187.0 ± 36.0	*P = 0.41*	202.2 ± 58.5	199.6 ± 45.9	*P = 0.80*
Mean N2 amplitude	11.3 ± 7.6	14.1 ± 6.5	*P = 0.16*	12.6 ± 9.9	12.0 ± 7.4	*P = 0.77*
Mean P2 amplitude	10.5 ± 4.3	10.4 ± 7.6	*P = 0.96*	15.1 ± 10.7	13.2 ± 9.4	*P = 0.57*
Mean N2-P2 amplitude	21.4 ± 8.9	22.4 ± 9.2	*P = 0.64*	25.2 ± 9.0	23.6 ± 13.0	*P = 0.50*

#### EEG results

A trendwise or significant increase in the theta band was observed following sham and active stimulation, respectively. Theta Fpz showed a trendwise increase after sham stimulation (*p* = 0.09), and theta Fz showed significant increase after sham stimulation (*p* = 0.04). After active stimulation, theta Fpz and Fz increased by trend (*p* = 0.09 and *p* = 0.09, respectively; Table 5).

## Discussion

This study aimed to explore the impact of prefrontal tDCS on pain in 16 MS patients. Active anodal but not sham tDCS showed significant analgesic effects on VAS_0−100_ pain ratings in the first 3 days and over 1 week after stimulation. These effects were further reflected by a significant decrease in the BPI total score and its interference subscale following only active stimulation. No relevant changes in BPI severity subscale were found after either stimulation conditions. Similarly, neither intervention had any effects on PREPs (amplitudes and latencies), ANT parameters, fatigue and mood scales. Lastly, a trendwise or a significant increase in the frontal theta activity was observed following active or sham stimulation, respectively.

### Effects on pain

The most prominent effect of tDCS treatment was analgesia. Such findings are of high interest and in line with previous studies. In fact, the DLPFC is known to have a crucial role in modulating pain (Lorenz et al., 2003). Interestingly, changes in pain perception following tDCS have been documented in several experimental protocols where anodal tDCS over the DLPFC increased the pain thresholds induced by electrical and heat stimuli in healthy volunteers (Boggio et al., 2008; Mylius et al., 2012).

As for the BPI scale, the fact that anodal tDCS over the prefrontal cortex ameliorated the interference but not the severity subscale might be accounted by the pathophysiologic mechanisms of pain. Actually, a pain matrix was conceptualized and includes three interacting networks (Garcia-Larrea and Peyron, 2013). A first order nociceptive network comprises the posterior operculoinsular area receiving spinothalamic projections. A second order network consists of the posterior parietal, prefrontal and anterior insular areas, and is responsible of the transition from cortical nociception to conscious perception. A third network is composed of the orbitofrontal, perigenual and limbic areas, by which the pain perception can be modified in function of expectations, emotions and beliefs. Therefore, our results suggest that tDCS could have acted on the second and third order networks of the pain matrix where the prefrontal cortex majorly contributes (Garcia-Larrea and Peyron, 2013).

### Effects on attention

Concerning attention, ANT variables did not significantly change after any of the tDCS interventions. In fact, we hypothesized that tDCS over the DLPFC could improve attentional capacities by modifying the sensitivity of stimulus-specific neural responses, in a way that enhances the top-down selection type or inhibits the bottom-up one; notably, by amplifying or inhibiting the activity of neurons which respond, respectively, to relevant or irrelevant stimuli (Desimone and Duncan, 1995). In accordance with our speculation, anodal tDCS over the left DLPFC have found to improve the attentional bias acquisition in one study (Clarke et al., 2014), and sustained attention in another one (Nelson et al., 2014). However, the lack of significant improvement of ANT parameters in our study can be explained by many facts. First, ANT is known to represent a complex task which measures three different attentional networks and hence involves several cortico-cortical and cortico-subcortical connections. Second, in contrast to previous tDCS studies in healthy subjects, MS patients are known to have a dysfunction in the attentional networks, notably in the alerting and/or the orienting ones (Urbanek et al., 2010; Crivelli et al., 2012; Omisade et al., 2012; Vázquez-Marrufo et al., 2014; Ayache et al., 2015) which might have prevented tDCS from causing any improvement. Third, our tDCS design might not have been optimal to ameliorate ANT variables. It is noteworthy that a lateralization in the attentional performance exists between left and right hemispheric structures (Corbetta and Shulman, 2002; Raz and Buhle, 2006; Lückmann et al., 2014). In fact, the left and right hemispheres are thought to be involved in phasic and tonic alertness, respectively (Raz and Buhle, 2006). In this model, the right DLPFC has an executory capacity which enables it to monitor the attentional performance and hence regulates it accordingly, while the right inferior parietal region seems to have a role in endogenous and exogenous alerting. In line with this topography, one study found that anodal tDCS over the right frontal cortex (F10 of the 10–10 EEG system of electrodes positioning) could improve the alerting network of attention (Coffman et al., 2012). Another study compared the effects of anodal tDCS over different cortical targets in terms of attentional improvement and showed significant effects following the stimulation of the right posterior parietal cortex (PPC) but not the left DLPFC (Roy et al., 2015). This suggests that unlike our design, which targeted the left hemisphere, stimulation of the right fronto-parietal structures might have better effects on attention. Fourth, our protocol consisted of performing ANT before the first stimulation session (D1) and after the last one (D3) of each block, but did not contain a “within-day control” of attention. This was based on the hypothesis that a tDCS-induced cumulative effect might result from the repetition of the stimulation sessions. However, three consecutive daily sessions might not be enough to induce such an effect, and therefore a within-day control for attention would have revealed better outcomes. Fifth, concerning the tDCS montage adopted in this study, the anode was placed at the left DLPFC while its reference electrode was the right supraorbital region. We should note that the chosen reference is not neutral. In fact the orbitofrontal cortex has an important role inemotional and cognitive processing, including the attentional processes to emotional stimuli. Therefore, applying a cathodal stimulation on that region might have impacted our results.

These data altogether suggest that future studies might benefit from comparing the effects of tDCS over different cortical areas (e.g., left/right DLPFC and left/right PPC), prolonging the duration of the stimulation period, adding a within-day control for attention, and adopting a different reference site, before drawing any conclusion.

### Effects on mood and fatigue

As for mood changes, although there are evidence regarding the antidepressant effects of anodal tDCS over the DLPFC (Kalu et al., 2012; Loo et al., 2012; Berlim et al., 2013; Brunoni et al., 2013), the lack of mood changes in our patients cohort can be attributed to the short stimulation period adapted in this study. In this perspective, depression studies have shown dose-dependent effects of tDCS in mood improvement that could extend over several weeks. Our results are supported by another study where no improvement in mood scores has occurred following five tDCS sessions over the left DLPFC (Saiote et al., 2014).

Concerning fatigue, we found no effects of tDCS on the MFIS scores. A lack of improvement in fatigue following tDCS has already been reported by two studies (Ferrucci et al., 2014; Saiote et al., 2014).

### Effects on neurophysiological parameters

#### PREPs

PREPs amplitudes were not changed following both active and sham tDCS. Although PREPs can correlate with the subjective pain sensation, it is commonly accepted that PREPs amplitude reflects the integrity of the spino-thalamo-cortical tract (Casey et al., 1996; Wu et al., 1999; Garcia-Larrea et al., 2002). In this view, most of the neuroimaging studies have demonstrated that MS patients with neuropathic pain had several demyelinating lesions within the spinal cord, brainstem, and thalamus (Seixas et al., 2014). Furthermore, PREPs amplitude is known to depend on the attentional level toward the stimulated limb (Garcia-Larrea et al., 2002), in this context, attentional dysfunction has been frequently reported in MS patients. Taken together, these disturbances might have prohibited tDCS from having any effect on PREPs parameters in our cohort.

#### EEG

As for the frontal midline theta activity, their increase following both stimulation blocks can be explained by different approaches. In fact, anxiety improvement has previously been correlated with the appearance of such waves following anxiolytic treatment (Mizuki et al., 1983, 1989). In addition, an emotionally positive state was found to be associated with theta power in frontal midline leads (Aftanas and Golocheikine, 2001). In this perspective, it might be possible that medical care itself, regardless of stimulation block type, had accounted for a subtle decrease in patients' anxiety, despite the absence of any significant changes in HADS scores.

## Limitations

It is important to note that, in our patients' cohort, the observed tDCS effects could have been influenced by antiepileptic treatments. The latter are known to inhibit long-term-potentiation induced neuroplasticity changes by blockade of voltage-gated ion channels (Nitsche et al., 2012). However, in most of the studies dealing with chronic neuropathic pain, patients commonly suffer from a severe pain which is resistant to multiple drugs. Particularly, the available tDCS studies for central neuropathic pain have included patients taking antiepileptics, antidepressants, and opioid analgesics (Fregni et al., 2006; Soler et al., 2010; Wrigley et al., 2013). Similar to our work, the authors asked their patients to keep their routine medications throughout the study period. Another limitation would be the lack of functional or structural neuroimaging data in our work. Future studies can benefit from combining imaging with tDCS application, in order to understand the mechanisms of action of tDCS on the studied brain networks, and to correlate the clinical response to gray matter and white matter pathologies (Chalah et al., 2015). In addition, the small sample size and heterogeneity of our MS cohort could have prohibited the emergence of any positive effect in terms of attention, mood and fatigue. Finally, MS-related symptoms can fluctuate during the course of the disease; such a fluctuation should be taken into account prior to the interpretation of any intervention results. In this context, our study could have benefited from testing the difference between baseline scores prior to tDCS and sham conditions. However, we believe that our inclusion criteria (e.g., the presence of a neuropathic pain since more than 3 months with an intensity greater than 40 on VAS; the absence of MS relapses since at last 2 months, a stable therapy since 1 month) might have restricted such an influence. We also controlled for the impact of some variables on attention, pain, and fatigue; namely by restricting the consumption of psychostimulants, and performing the stimulation block at the same time during the day, with no blocks occurring in summer.

## Author contributions

MA, MS, DD, and AC: enrolled patients from the neurology department of Henri Mondor Hospital. SA, UP, and MC: performed the stimulation sessions and collected the data. SA and JL: designed the study, developed the methodology, interpreted the data and wrote the final draft. MC and UP: wrote the first draft. TA and AB: analyzed the EEG data.

### Conflict of interest statement

AC gave expert testimony for CSL Behring, Novartis, received grants from Biogen, Novartis, CSL Behring, GE Neuro, Octapharma, and gave lectures for Genzyme. The remaining authors declare that the research was conducted in the absence of any commercial or financial relationships that could be construed as a potential conflict of interest.
